# Effect of pocket irrigation with antimicrobial on prevention of pacemaker pocket infection: a meta-analysis

**DOI:** 10.1186/s12872-017-0689-9

**Published:** 2017-09-30

**Authors:** Feng-Guang Kang, Pei-Jian Liu, Li-Yi Liang, Yong-Qing Lin, Shuang-Lun Xie, Yi He, Bao-Shan Liang, Hai-Feng Zhang, Yang-Xin Chen, Jing-Feng Wang

**Affiliations:** 1 ShunDe Hospital of Guangzhou University of Chinese Medicine, Foshan, Guangdong 528333 People’s Republic of China; 2 Department of Cardiology, Sun Yat-sen Memorial Hospital, Sun Yat-sen University, Guangzhou, Guangdong 510120 People’s Republic of China; 3 Laboratory of Cardiac Electrophysiology and Arrhythmia in Guangdong Province, Guangzhou, Guangdong 510120 People’s Republic of China

**Keywords:** Cardiac implantable electronic devices, Pocket irrigation, Pocket Infection, Meta-analysis

## Abstract

**Background:**

The presence of cardiac implantable electronic devices (CIEDs) pocket infection is difficult to treat, causing serious clinical outcomes, but little is known for prevention. Results from some studies suggested that pocket irrigation could reduce infection while others showed conflicting results. We pooled the effects of pocket irrigations on the prevention of pocket infection by meta-analysis methods.

**Method:**

Relevant studies published before June, 2017 were retrieved mainly by the computer-based search of PubMed, Cochrane, EMBASE, Web of Science, Chinese BioMedical, Global Health and BIOSIS Previews databases. Estimations of relative ratios (RRs) and 95% confidence intervals (95% CIs) were pooled. Subgroup analyses according to potential key factors affecting the effects were conducted, which was confirmed by meta-regression. Sensitivity analysis and test for publication bias were also performed.

**Results:**

We identified 10 studies providing data of 5467 patients receiving CIEDs implantations. Pooled infection rates were 1.48 and 3.49% respectively for medication and saline irrigation groups. Meta-analysis showed that medication irrigation conferred protection to pocket infection (RR = 0.44, 95% CI: 0.31-0.63). Subgroup analysis showed that antibiotics, rather than non-antibiotics (antiseptics) exerting the protection. The first and second lines antibiotics against *staphylococcus aureus*, which is the main pathogen for pocket infection, were both effective (RR = 0.42, 95% CI: 0.24-0.75 and RR = 0.34, 95% CI: 0.20-0.58 respectively for first line and second line therapies). Meta-regression revealed that region and class of irrigation medication completely explained the variance among studies and implied that effects of region were masked by medication types. Sensitivity analysis did not showed any significant change of the result and publication bias were not statistical significance.

**Conclusion:**

Pocket irrigation with antibiotics were effective for reducing pocket infection and should be encouraged in CIEDs implantation.

## Background

Cardiac implantable electronic devices (CIEDs) mainly include pacemaker, implantable cardiac defibrillator (ICD), cardiac resynchronization therapy (CRT). Of these, pacemakers are the most common and effective way to treat bradycardia arrhythmia (pacemaker), while ICD and CRT are effective strategies to prevent sudden death and improve heart failure, respectively. The number of CIEDs implantations have increased significantly during the past decade. There were 560,000 CIEDs implanted in the United States every year [1, 2]. Accordingly, the number of infectious complications attributed CIEDs implantation also increased dramatically [1, 3]. Subcutaneous pocket infection, which is mainly due to *staphylococcus aureus* [4], is the most serious infectious complications [5, 6]. The reported incidence of these CIEDs-related infections has been ranging from 0 to 12.6% in studies [3, 7, 8], mostly ranging from 1 to 7% [9].

Among all the complications, pacemaker/ICD/CRT pocket infection remains a serious, and even, potentially life-threatening complication [8]. Once pocket infection occurred, it will prolong hospital stay, increase medical cost, especially when a removal of the entire system with subsequent re-implantation is needed [10–12]. Moreover, the increased incidence of infectious complication is associated with substantial elevated morbidity and mortality [8, 13, 14].

Although there are data supporting systemic use of antibiotic for preventing infection during and after CIEDs implantations, many employ other accompanying strategies for further prevention [2, 15]. One of the strategy is pocket irrigation with antimicrobial solutions, although there is no conclusive evidence demonstrating its benefit. Indeed, results from individual trials were conflicting and not convincing. For example, results from Lakkireddy and colleagues showing an infructuous effect of pocket irrigation while other claimed a reduced rate of infection [16, 17]. To summary current evidence and draw a plausible conclusion, we performed a meta-analysis of available trials to re-evaluate the effectiveness of pocket irrigation with antimicrobial agents in reducing pocket infection during CIEDs implantation.

## Methods

### Literature search and study selection

All studies reporting the effects of pocket irrigation during CIEDs implantation published before June 2017 were identified by the comprehensive computer-based search of PubMed, Cochrane, EmBase, Web of Science, Chinese BioMedical, Global Health and BIOSIS Previews databases. The following terms were used for search: pacemaker, cardiac implantable device, VVI, DDD, implanted cardiac defibrillator, ICD, cardiac re-synchronization therapy and CRT, which were combined with pocket, irrigation, and infection. Hand searches for related articles were also performed. All the searches were conducted without language restriction. Reference lists of the retrieved articles were also reviewed to ensure to no eligible study missed.

For inclusion, procedure of the implantation had to be described properly to ensure that no operative factors leading to infection. Exclusion criteria were as follows: 1) no recording infectious rates between groups; 2) non-medication treatments of the pocket; 3) including pericardial lead implantation; and 4) sample size less than 100.

### Data extraction and quality assessment

Two investigators (Kang FG and Zhang HF) extracted data independently. All the data were extracted using a standardized data-collection form. Information was recorded as follows: last name of the first author, year of publication, geographical location, study design, agents for irrigation, time from implantation to infection, duration of the follow-up period and number of participants.

The quality of enrolled studies were also assessed by the same investigators and the following elements were considered [18]: study design, characteristics of the studied population, assessment of outcome, duration of follow-up, and statistical control for potential confounding factors. Any disagreement were resolved by a discussion. All the data were extracted from published results and written informed consent for participation was not applicable.

### Statistical analysis

Meta-analysis was performed as our previous report [19, 20]. In brief, heterogeneity of effect size across studies was quantified by the *I* ^*2*^-statistic and tested by a Cochrane *Q*-test with a significance level of *P* < 0.1, rather than 0.05 [21]. Pooled effect size was estimated by Mantel-Haenszel fixed-effects model if no significant heterogeneity existed. Otherwise, the DerSimonian-Laird random-effects model was adopted. Potential publication bias was assessed by Egger’s test and Begg’s funnel plot was produced [22].

To further investigate the effects of pocket irrigation during CIEDs implantations, subgroup analyses according to study designs, classes of medications, time from implantation to infection, and geographical locations were performed. Meta-regression using restricted maximum likelihood estimation method was adopted to explore sources of heterogeneity and to confirm results from subgroup analyses [23]. *P*-values were adjusted with the Monte Carlo method (permute = 100) if more than 3 co-variables enrolled in the regression model to reduce type I error [24]. Finally, a sensitivity analysis, which investigated the influence of a single study on the overall risk estimated by omitting one study in each turn, was used to test the stability of the pooled results.

The study was performed in accordance with the PRISMA statement [25]. All analyses were performed by using STATA version 12.0 and graph of quality assessment was produced by Revman 5.3.

## Results

### Eligible studies

**Fig. 1:**
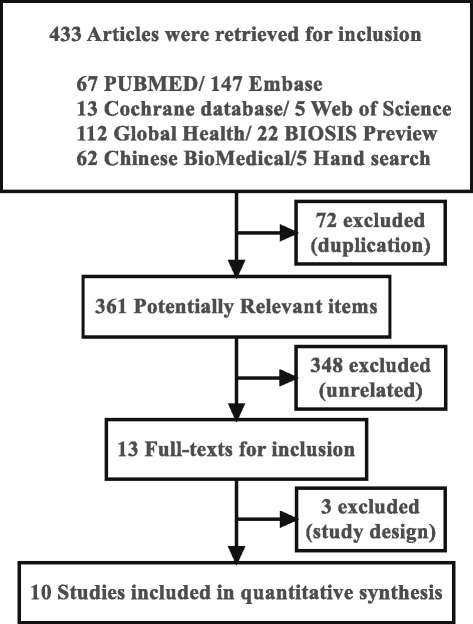
Systematic search and screening process of included trials

With separated search strategy in each database, a total of 433 articles that potentially pertinent were retrieved. By reviewing titles and abstracts, irrelevant studies, case reports and reviews were excluded. Finally, 13 studies were identified for further considerations. Of the these, 3 studies were further excluded mainly due to an absence of control group (two studies) and comparing short term and long term usage of antibiotics (one study). Therefore, ten studies including 5467 patients (3117 in irrigation group and 2340 in control group) completely met the inclusion criteria, which were used in the later analysis [16, 17, 26–33]. The flow diagram of searching and screening publications were listed in Fig. 1.

### Study characteristics

Characteristics of the included studies were presented in the Table 1. Overall, enrolled trials were published from 2003 to 2017, including American and Chinese studies, and ranging from 118 to 2654 in sample size. Most of the studies used antibiotics and one study used povidone-iodine to irrigate the pocket [16]. Of the trials using antibiotics, the third generation of cephalosporin was used in three studies, dcefazolin and gentamicin were used in two studies, clindamycin and azithromycin were used in only one study. Besides, povidone-iodine was used in one study (Table 1). Two studies were retrospective in design and the rest were prospective ones (Table 1).

**Table 1:** Characteristics of the included studies

Study	Location	Study design	Gender (*n*, male)	Agent	Follow-up period	Samle size	Treatment	Control
							Infection/Total	Infection/Total
Lu et al. 2003 [28]	China	Prosepctive	72	3rd cephalosporin	7 years	118	0/61	0/57
Guo et al. 2005 [27]	China	Prosepctive	101	Cefazolin	2 years	178	2/90	0/88
Lakkireddy et al. 2005 [16]	America	Retrospective	1718	Povidone-iodine	8 years	2564	10/1359	8/1205
Xia et al. 2007 [29]	China	Prosepctive	66	Gentamicin	3 years	122	1/63	1/59
Zhou et al. 2010 [32]	China	Prosepctive	155	Cefoperazone/ Tazobactam	1 month	268	2/84	9/84
Wang et al. 2015 [17]	China	Prosepctive	70	Cefazolin	>4 weeks	116	2/58	8/58
Yang 2015 [30]	China	Retrospective	931	Amikacin/ Gentamicin	7 years	1572	15/1133	16/439
Zhang et al. 2016 [31]	China	Prosepctive	77	Cefatriaxone	6 months	146	5/73	21/73
Lakshmanadoss et al. 2016 [33]	America	Retrospective	134	Clindamycin phosphate	1 year	327	2/118	2/209
Chen et al. 2017 [26]	China	Prosepctive	95	Azithromycin	1 year	156	7/78	17/78

**Fig. 2:**
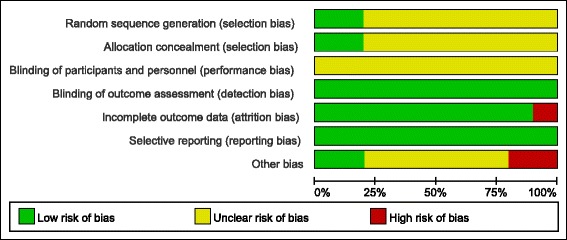
Quality assessments of included studies

All the included studies were low in detection and attrition bias, while most studies were with low in attrition bias and more than a half of the studies were with low to unclear bias in selection, performance and other bias (Fig. 2).

### Main analyses

#### Pocket irrigation with anti-biotics was effective to reduce pocket infection

**Fig. 3:**
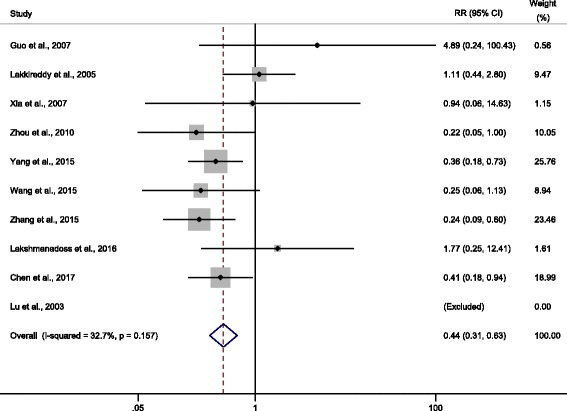
Meta-analysis of all the included studies on protective effects conferred by pocket irrigation. Estimated effect size was derived by Mantel-Haenszel fixed-effects model and heterogeneity test *P*-value was calculated by Cochrane *Q*-test. Size of the box represented weight of the study on the over-all results

Overall, pocket infection rate was 1.48% (46/3117) in the irrigation group and 3.49% (82/2350) among the controls. Pooled analysis including all the studies was firstly performed and results from fixed-effects model showing a beneficial effect of pocket irrigation, reducing 56% infectious rate (OR = 0.44, 95% CI: 0.31 to 0.63, Fig. 3). Mild to moderate heterogeneity was observed among studies (*I* ^2^ = 32.7% and *Q*-test *P* = 0.16). This result showed that the initial analysis support a protective role of pocket irrigation using medications.

**Fig. 4:**
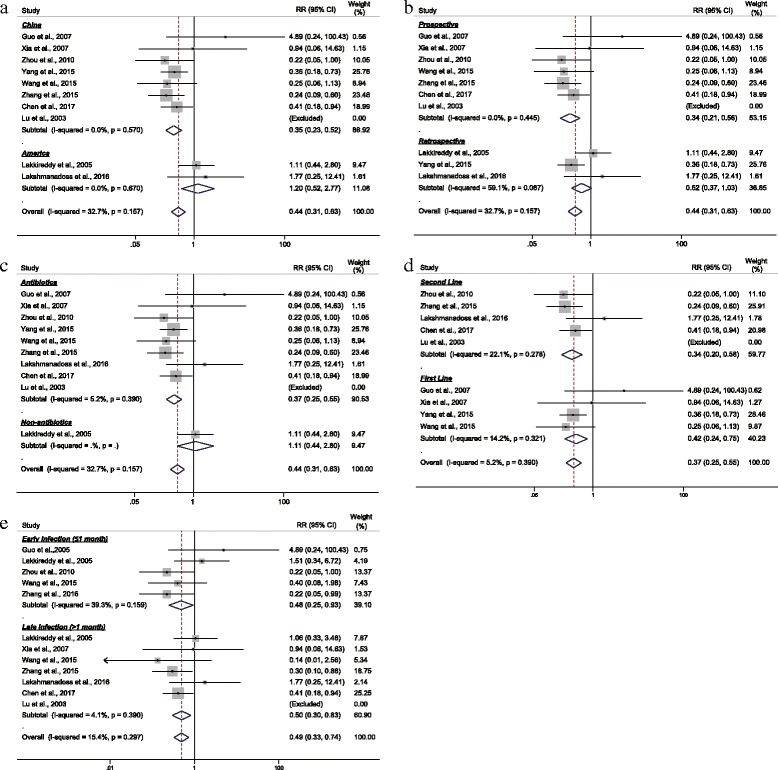
Subgroup analyses according to region (**a**), irrigation medications (**b**), study design (**c**), and first/second line therapy for *staphylococcus aureus* (**d**) and early/late infection (**e**). Methods used and meaning of symbols were the same as Fig. 3

Despite the above analysis did not yield much heterogeneity, we continued to perform subgroup analysis to further investigate factors that may influence the magnitude of effect size. Subgroup analysis results were presented in Fig. 4. As indicated, the protective effects of pocket irrigation were dismissed in American studies and non-antibiotics cohorts (Fig. 4a and b). Similarly, results from prospective trials differed from those of retrospective ones (Fig. 4c). Both first line and second line antibiotics therapies against *staphylococcus aureus*, which is the leading pathogen of pocket infection, were effective but more benefits were observed in sencond line therapy (Fig. 4d). Pocket irrigation reduced both early (within 1 month) and late (longer than 1 month) infections, but with a slightly larger effect size in protecting early infection (Fig. 4e). All these results confirmed the protective role of antibiotics, irrespective of class of medications.

#### Irrigation with non-antibiotics did not reduce pocket infection

The above analyses found that results from the American cohort (two studies), retrospetive cohort (three studies) and non-antibiotics cohort (one study) did not support the protective role of pocket irrigation. Notably, there was a same study using non-antibiotics in all of these cohorts.

**Fig. 5:**
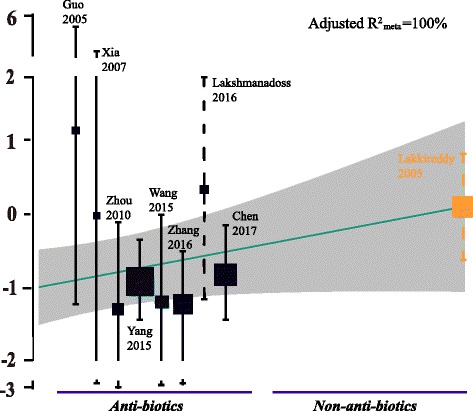
Meta-regression using interaction between region and class of medication (antibiotics/non-antibiotics) used for irrigation. The interaction explained almost all of the variance among enrolled studies. Meta-analysis was performed using restricted maximum likelihood estimation. Size of the box represented study weight. Symbols represents indicated RRs and 95% CIs of the study and dotted line indicated the American study. Orange symbols indicated study using non-antibiotics (povidone-iodine). Gray shading area represented 95% CIs of the regression line

To confirm the above results from subgroup analyses, we performed meta-regression to seek the potential co-variables on protective effects conferred by irrigation. Meta-regressions using mean age, region, study design, including patients receiving ICD (or CRT) or not, antibiotics/non-antibiotics, first/second line therapy against *staphylococcus aureus*, and early/late infections as co-variables independently were done. Results indicated that age, study design, including CRT or ICD patients, first/second line therapy for staphylococcus aureus, or early/late infections were not the source of heterogeneity (mean age: *P* = 0.34; study design: *P* = 0.24; CRT or ICD included: *P* = 0.72; first/second line therapy for *staphylococcus aureus*: *P* = 0.18; early/late infections: *P* = 0.33). However, both region and antibiotics/non-antibiotic may account for heterogenetity (region: *P* = 0.03; antibiotics/non-antibiotics: *P* = 0.08). Both of the two co-variables completely account for the intra-studies variance (both adjusted R^2^_meta_ = 100%). Because one of the study using non-antibiotics was the American one [16], we therefore further generated an interaction variable between region and antibiotics/non-antibiotics used, which was later used in meta-regression. Results revealed that effects of this interaction (region and antibiotics/non-antibiotics) were exactly the same as using non-antibiotics alone as co-variable (both regressions OR = 2.99, *P* = 0.08, Fig. 5). Therefore, the meta-regression results implied that the significant influence manifested by region may be actually masked by antibiotics/non-antibiotics used in studies.

#### Sensitivity and publication bias

**Fig. 6:**
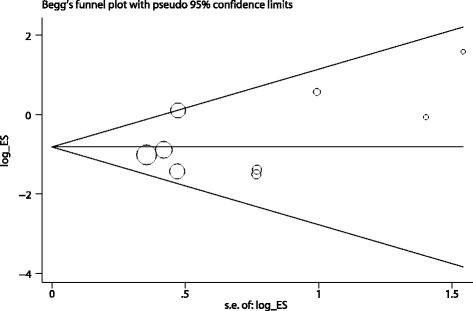
Publication bias of included studies. *P*-value was derived by Egger’s test. Size of the circle indicated study weight

Furthermore, we performed sensitivity analysis to examine the intensity of the conclusion and results found that the protective effects of pocket irrigation were not influenced, which remained to be significant omitting any of the included study. These results implied that the protective effects of pocket irrigation were stable and robust. Publication bias determined by Begg’s test did not showed a significant bias (Kendall’s Score = 12, *P* = 0.21, Fig. 6).

## Discussion

Pocket infection is the most serious complication of CIEDs implantation and current evidence on the effects of pocket irrigation is inconsistent. To summary available information in this field, the meta-analysis of 10 studies involving 5467 participants supported a protective effect of antibiotics pocket irrigation, irrespective of antibiotics classes. Compared with the use of saline, the incidence of pocket infection was reduced by about 59% with antibiotic irrigation.

Implantations of CIEDs have increased over the past decades, despite implantation technique improvement, the infection rate has increased dramatically [2, 8]. Pocket infection may be due to the following causes [34]: (1) Pacing leads and pulse generators are exposed to the air before implantation, which may cause airborne pathogens; (2) To the human body, the implant is a metallic foreign body which has a rough surface, making it prone to bacteria growth; (3) Operation duration, which is greatly affected by the individual condition of the patient and the technique of the operator; (4) The ambient air quality in the operating room, which may not be sufficient to reach the real sterile in some time; (5) Patients receiving CIEDs implantation are mostly aged ones, who could have various co-existing diseases, making them more prone to be infected. It is obvious that most of the reasons could not be completely avoided and strategies to prevent pocket infect becoming crucial.

Besides systematic application of antibiotics, little effective strategy is known to reduce pocket infection. Therefore, screening and confirming positive methods to reduce pocket infection is of great importance. Pocket irrigation may be a promising strategy for reducing pocket infection but debate on this issue is persistent during the decade [7, 35]. Indeed, pocket infection, despite increasing, is not with high prevalence, which result in a need of large sample size to give a statistical significance. It may be difficult for a single study to reach such a large sample size. Because of the inconsistent results of the trials, current guideline did not give a recommendation on pocket irrigation to reduce pocket infection [36]. Therefore, pooled analysis of existing information in this field becoming important and emergent. The current study pooled all the data on pocket irrigation and showed a benefit of reducing infection, irrespective of class of antibiotics, which robustly supported that the use of antibiotics for pocket irrigation should be encouraged. Indeed, an survey of 2092 electro-physiologists in more than fifty countries believed that the use of anti-microbial agents for pocket irrigation could reduce infection of CIEDs implantation [35]. Thus, the current study provide conclusive evidence supporting this view point.

The same important is to identify the most effective drugs for irrigation. As known, the most common bacteria causing pocket infection is *staphylococcus aureus* [12, 36], which accounts for about 60 to 80% of the pathogens in infection [6]. For the treatment of *staphylococcus aureus*, cefazolin and gentamicin were recognized as the first line therapy while others were believed as the second line [6]. As indicated in the present study, the second line therapy for *staphylococcus aureus* was more effetive despite the first line therapy also conferred protective effects. Therefore, when choosing the medication for pocket irrigation for whom are proning to be infected, drugs of the second line therapy may be considered first. Besides, drug resistance of the bacteria in their own center should also be taken into consideration. For example, in south of China, cefazolin is the most frequently used while the third generation of cephalosporin and lincosamide were adopted in north of China and the USA respectively [31, 33].

Some limitations of the current study should be noted. Firstly, despite subgroup analysis of RCTs/non-RCTs also showed an ineffectiveness of retrospective studies, meta-regression did not support study design as the variable for variance. Of note, one of the retrospective study using non-antibiotics, which was shown to be the source of the heterogeneity. Therefore, it is unknown whether the retrospective study did not support the adoption of pocket irrigation in nature, or like co-variable of region, which is actually masked by the use of non-antibiotics in the retrospective study. Secondly, in-sufficient data restricted further analysis according to different kinds of CIEDs (VVI/DDD/ICD/CRT/CRT-D) implantations, which might also be an important issue since patients receiving ICD/CRT/CRT-D were largely heart failure or ventricular arrhythmia survivors, who may be much more complicated and have distinct properties concerning with pocket infection.

## Conclusion

The current study demonstrated significantly protective effect on preventing pacemaker pocket infection with antibiotics pocket irrigation, irrespectively of classes of antibiotics, which robustly supported regular use of antibiotics for pocket irrigation during CIEDs procedure. As well as a need for large sample size confirming this conclusion in prospective studies with well control of bias, there is also an urgent need for screening of most effective medication, which together contribute to the reduction of pocket infection.

## Abbreviations

CI: Confidence interval
CIEDs: Cardiac implantable electronic devices
CRT: Cardiac resynchronisation therapy
CRT-D: Cardiac resynchronisation therapy with a defibrillator
ICD: Implantable cardiac defibrillator
RR: Relative ratio
